# Sexual reproduction and genetic polymorphism within the cosmopolitan marine diatom *Pseudo-nitzschia pungens*

**DOI:** 10.1038/s41598-020-67547-9

**Published:** 2020-06-30

**Authors:** Jin Ho Kim, Penelope Ajani, Shauna A. Murray, Joo-Hwan Kim, Hong Chang Lim, Sing Tung Teng, Po Teen Lim, Myung-Soo Han, Bum Soo Park

**Affiliations:** 1 Department of Life Science, College of Natural Sciences, Hanyang University, Seoul, 04763 Republic of Korea; 2 Risk Assessment Research Center, Korea Institute of Ocean Science and Technology, Geoje, 53201 Republic of Korea; 3 DNA Analysis Division, National Forensic Service, Seoul, 158-707 Republic of Korea; 4 Climate Change Cluster, University of Technology, Sydney, 2007 Australia; 5 Regal City College, 93150 Kuching, Sarawak Malaysia; 6 Faculty of Research Science and Technology, University Malaysia Sarawak, 94300 Kota Samarahan, Malaysia; 7 Bachok Marine Research Station, Institute of Ocean and Earth Sciences, University of Malaya, 16020 Bachok, Kelantan Malaysia; 8 Marine Ecosystem Research Center, Korea Institute of Ocean Science and Technology, Busan, 49111 Republic of Korea

**Keywords:** Experimental evolution, Speciation, Evolutionary ecology

## Abstract

Different clades belonging to the cosmopolitan marine diatom *Pseudo-nitzschia pungens* appear to be present in different oceanic environments, however, a ‘hybrid zone’, where populations of different clades interbreed, has also been reported. Many studies have investigated the sexual reproduction of *P. pungens,* focused on morphology and life cycle, rather than the role of sexual reproduction in mixing the genomes of their parents. We carried out crossing experiments to determine the sexual compatibility/incompatibility between different clades of *P. pungens*, and examined the genetic polymorphism in the ITS2 region. Sexual reproduction did not occur only between clades II and III under any of experimental temperature conditions. Four offspring strains were established between clade I and III successfully. Strains established from offspring were found interbreed with other offspring strains as well as viable with their parental strains. We confirmed the hybrid sequence patterns between clades I and III and found novel sequence types including polymorphic single nucleotide polymorphisms (SNPs) in the offspring strains. Our results implicate that gene exchange and mixing between different clades are still possible, and that sexual reproduction is a significant ecological strategy to maintain the genetic diversity within this diatom species.

## Introduction

At least 20 different species concepts have been formulated^1–3^, including those based primarily on the identification of unique morphological features, those based on the sexual compatibility, and those based on phylogenetic or other evolutionary or ecological features^4^. Phytoplankton species are generally delineated on morphological characteristics, however phylogenetic studies have shown that molecular genetic data can indicate species boundaries which differ to those postulated based solely on morphological features. Those species which cannot be distinguished on morphological criteria alone are known as ‘cryptic’ species^5,6^. There are many cryptic or pseudo-cryptic species in plants and animals as well as micro-organisms including phytoplankton species^7–11^. Among many species concepts, an application of the biological species concept can be used to clarify species delineation in heterothallic species, as mating compatibility information can explain the discrepancies within microorganism species which are difficult to distinguish based on molecular genetic or morphological features^1,12^.

Phenomena such as meiosis and crossing over, mutations, lateral gene transfer maintain genetic diversity which potentially allows species to adapt to various conditions^13^. Genetic variability can arise through several mechanisms; firstly, by migration and genetic recombination through sexual reproduction of co-occurring populations or genotypes^14,15^; secondly, by mutations^16–18^; and thirdly, by gene transfer^19,20^. Interbreeding is significant in the life and evolution of diatoms. Diatoms have a unique life-cycle in which they undergo continued vegetative cell division, resulting in cell size decrease, followed by cell size recovery through sexual reproduction^21–24^. Hybrids might contribute adaptive variation to existing species^25,26^, and provide an impetus for new species by making the genetic recombinant hybrids^27,28^. There has not been much research on genetic polymorphism due to sexual reproduction. One of the reasons is that sexual reproduction in diatoms is hard to observe in the field^29–33^, because sexual reproduction occurs every few years, but only takes 2–4 days to complete the process^13,24,34^.

The cosmopolitan diatom *Pseudo-nitzschia pungens*, is a good model organism to examine the evolution and species concept in marine diatoms. This diatom is distributed globally and is highly diverse^35–39^. This species contains three genetically distinct groups (clade I, II and III), based on sequences of ribosomal DNA (rDNA) internal transcribed spacers (ITS) region^35^, and three clades of *P. pungens* characterized into three morphological varieties according to its differences of the cingular band structure. Clade I is comprised of *P*. *pungens* var. *pungens*, clade II comprises both *P. pungens* var. *pungens* and *P. pungens* var. *cingulata*, while clade III comprises both *P*. *pungens* var. *pungens* and *P. pungens* var. *aveirensis*^35,40–44^. Biogeographically, clade I is distributed globally in temperate zones, clade II is only found along the eastern North Pacific coasts, and clade III is reported from tropical warm-temperate areas^35,36,42,43^. Their distinct physiological characteristics formed by specific habitat environments have been reported^42^.

*Pseudo*-*nitzschia* species are generally heterothallic, meaning they require another mating type (e.g. PNp+ or PNp−) for sexual reproduction. Many sexual reproduction studies have been conducted using this genus to examine diatom reproduction and life cycles^22–24,33,45^, yet there are few studies which explore the role of sexual recombination in maintaining genetic diversity^7,46^. A few isolates of *Pseudo*-*nitzschia*, which are regarded as offspring from parents of different genotypes (based on the ITS sequencing), have been found in natural seawater^40,47^. To date, two hybrid zones of *P. pungens* have been found in the world; the first is Puget Sound, eastern North Pacific (for clades I and II)^40^, and another is the Korean coasts near the Yellow and East China Sea (for clades I and III)^48^. Several genetic recombinant cells of *P. pungens* have been found in Puget Sound as indicated by novel mixed sequences patterns (clades I and II) in the ITS region, however, it is still unclear whether or not these are the result of recent divergence or intraspecific hybridization^40^. Furthermore, sexual compatibilities are not verified between clade I and III, and between clade II and III. The aim of the present study is to specifically test sexual compatibility among the three clades of *P. pungens*, and describe the genetic and morphometric characteristics of resultant offspring to explore the gene flow between genetically diverged populations, and the genetic polymorphisms within species. These experimental evidences will assist in the better understandings of biological species boundary and evolutionary strategies to maintain the genetic diversity for this cosmopolitan diatom species.

## Results

### Sexual compatibilities and offspring establishment

*Pseudo-nitzschia pungens* is a heterothallic species^22^. Strain bearing auxospores were assigned to mating type PNp−, while strains successfully mating with a PNp− were assigned to mating type PNp+. Assignment to either PNp− or PNp+ was possible when the differences in cell size between parent cultures was enough to be estimated by visual microscopic inspection. Successful mating results were observed within same clades, and between clades I and III and clades I and II strains without exception, but not between clades II and III although strains belong to opposite mating-types (Table 1). Among the clade I strains, sexual reproduction occurred under all temperature conditions (15, 20 and 25 °C), however no events occurred between clades I and III at 15 °C, and only at 20 °C between clade I and II (Table 1). Four offspring strains (HYOFA5, HYOFB6, HYOFB7 and HYOFE5) were established between clade I and III strains (HY40E5 and Pnsb109) for further experiments. The success rate to establish the strain was 33.3% (5/15). The strain establishing failed despite interbreeding was successful in several co-cultivation sets (Table 1).

**Table 1:** Results of mating experiments using *Pseudo-nitzschia pungens* strains of different clades. The experiments with all combinations of *P. pungens* strains were conducted at 20 °C , and we additionally examined whether or not sexual reproduction occurred under different temperature condition (15 °C and 25 °C) using some positive combinations; the asterisks (^+^) were marked at these positive combinations. The simple O without asterisk indicates that mating experiment was only conducted at 20 °C, and it is unclear whether or not sexual reproduction occurs at different temperatures (15 °C and 25 °C).

Clade	Strain	Mating-type	HY38B2	HY40E5	HY40C3	HY48D2	Pnpng1	HY47B2	HY47B3	HY47B5	Pnsb109	HY29B9	HY48C9
I	HY38B2	PNp+	–										
	HY40E5	PNp+	X	–									
	HY40C3	PNp−	O^+++^^*S*^	n.c	–								
	HY48D2	PNp+	X	X	n.c	–							
	Pnpng1	PNp−	O^+++^^*S*^	n.c	X	O^+++^	–						
II	HY47B2	n.d	n.c	n.c	n.c	n.c	n.c	–					
	HY47B3	PNp+	X	X	O	n.c	O	O^***S***^	–				
	HY47B5	PNp+	X	X	n.c	X	O^+^	O	X	–			
III	Pnsb109	PNp−	O^++^	O^++^^*S*^	X	O^++^	X	X	X	X	–		
	HY29B9	PNp+	X	n.c	n.c	X	O	X	X	X	O^***S***^	–	
	HY48C9	PNp−	O	O	X	n.c	n.c	X	X	X	n.c	n.c	–

O: sexual reproduction occurred.

^+++^: sexual reproduction occurred at three temperatures (15, 20, and 25 °C), ^++^: at two temperatures (20 and 25 °C), ^+^: at 20 °C only.

X: sexual reproduction absents in mixed culture.

–: sexual reproduction absents in monoclonal culture.

^*S*^: establishment of strain with an initial cell of offspring.

n.c: mating experiment not conducted.

n.d: mating type not determined.

PNp+ and PNp−: mating types determined by a method of Chepurnov et al.^22^.

### Size threshold and sexual ability of offspring

**Figure 1:**
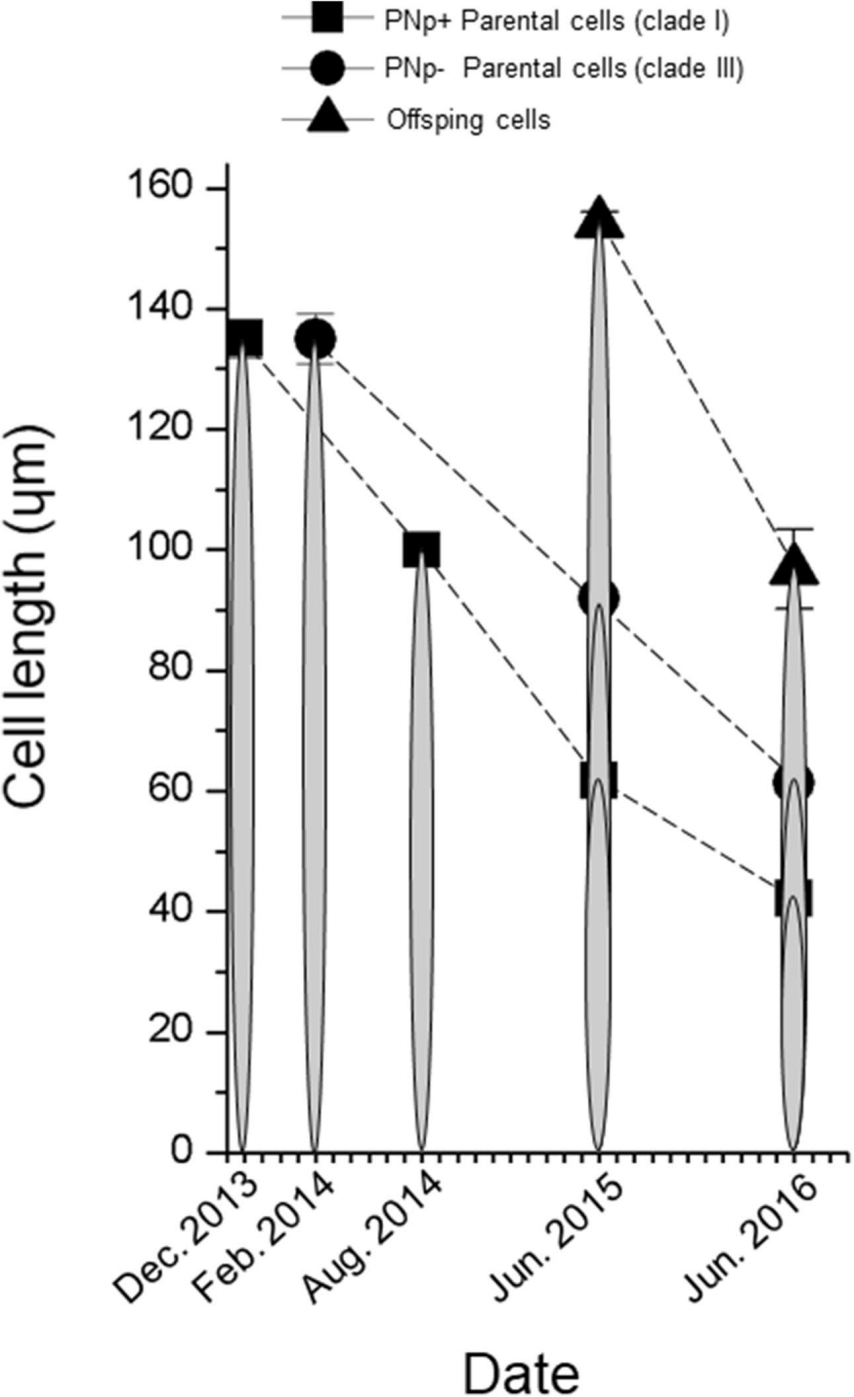
The mean valve lengths (n = 11) of parents (HY40E5; clade I and Pnsb109; clade III) and four offspring strains from date of establishment.

The valve length of each parent strain, when mating experiments were conducted, was 135 ± 3.2 and 135 ± 4.2 μm for HY40E5 (clade I) and Pnsb109 (clade III), respectively (Fig. 1). The valve length of parental strains gradually decreased and their lengths measured 61.7 ± 1.6 μm and 92.0 ± 1.5 μm (June 2015) when the mating experiments began (Fig. 1). The mean valve length of four offspring strains was 154.2 ± 1.7 μm when they were established (Fig. 1). Immediately after establishment of offspring strains, mating experiments were further conducted between offspring and parental strains, however no sexual reproduction was observed. After 1 year (in June 2016) sexual reproduction was observed between offspring and parental strains, as well as between offspring strains (Table 2). At this time, the mean valve lengths of parents were 42.2 ± 1.7 (HY40E5) and 61.3 ± 1.7 μm (Pnsb109) and the mean valve lengths of the four offspring strains were 96.8 ± 6.6 μm (Fig. 1).

**Table 2:** Results of mating experiments between and amongst offspring and parent strains.

Clone	Generation	Mating-type	HY40E5	Pnsb109	HYOFA5	HYOFB6	HYOFB7	HYOFE3
HY40E5	Parent	PNp+	–					
Pnsb109	Parent	PNp−	O	–				
HYOFA5	Offspring	PNp−	O	X	–			
HYOFB6	Offspring	PNp+	X	O	O	–		
HYOFB7	Offspring	PNp+	X	O	O	X	–	
HYOFE3	Offspring	PNp−	O	X	X	O	O	–

O: sexual reproduction occurred in mixed culture.

X: sexual reproduction absents in mixed culture.

–: sexual reproduction absents in monoclonal culture.

PNp+ and PNp−; mating types were determined by a method of Chepurnov et al.^22^.

### Genetic and geographic distances between parents

**Figure 2:**
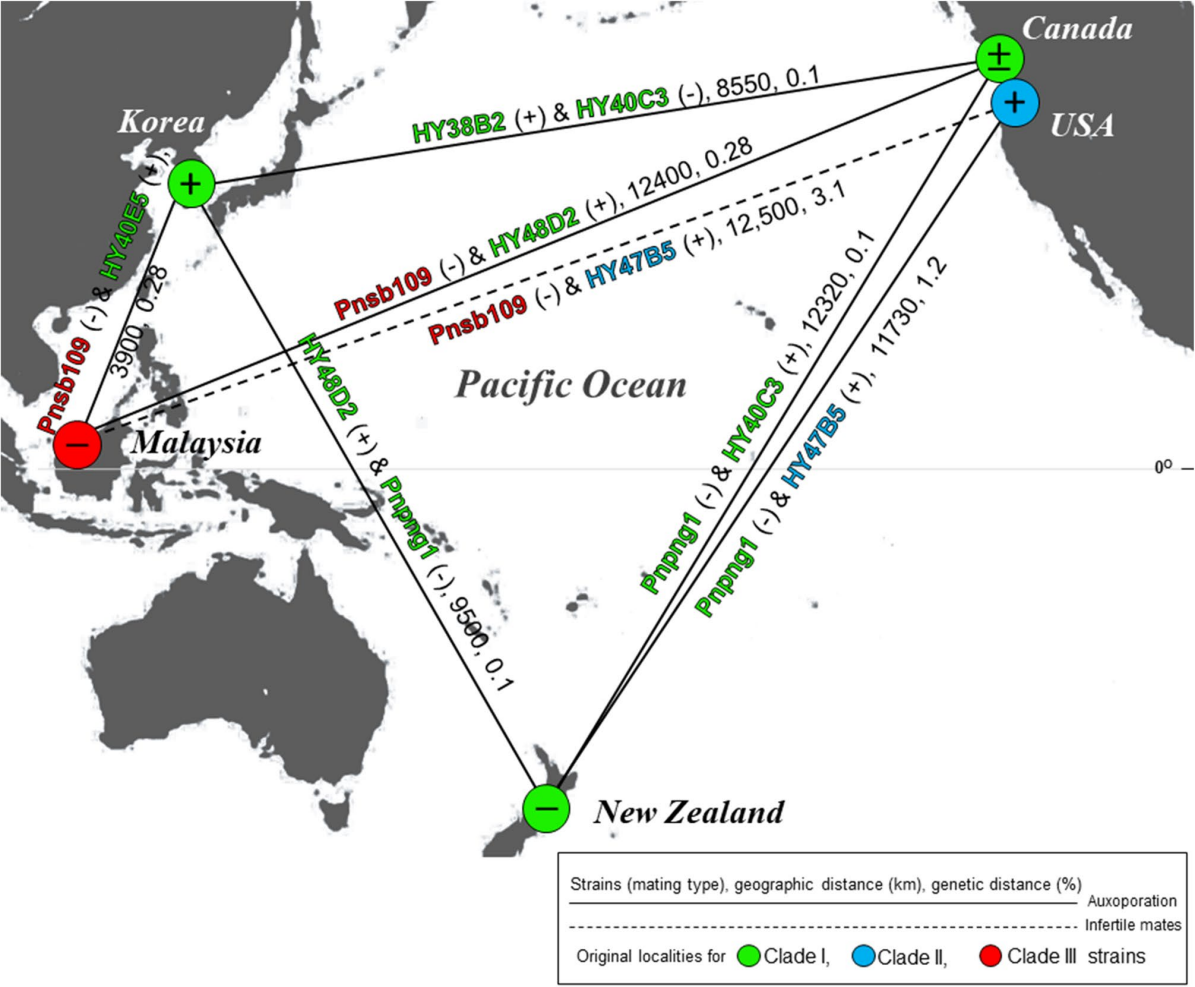
The results of mating experiments between *Pseudo-nitzschia pungens* strains belonging to three clades, and the geographical (km) and genetic distances (%) between parent strains. Each line represents the successful auxosporation while dotted line indicates infertility. Each geographic and genetic distance is presented on the lines of each mating pair. The mating types, + (PNp +) and − (PNp−) are determined by method of Chepurnov et al*.*^22^*.*

In total 258 base pairs of complete ITS2 were amplified and aligned. Evaluated genetic distances between clade I and II strains were 1.2%, between clade I and III strains were 2.8% and between clades II and III was 3.1% (Fig. 2; Supplementary Table S1). Among clade I strains, the genetic distance was less than 0.1%. Sexual incompatibility was not correlated with geographic distances between the sites of isolation of the parent strains. Regardless of geographic distances, sexual reproduction was possible between clades I and III, and clades I and II. The origins of two strains, HY47B5 (clade II) and Pnsb109 (clade III), were geographically distance (12,500 km) and sexual event did not happen, but it did happen between HY48D2 (clade I) and Pnsb109 (clade III) which had a similar geographic distance (12,400 km) (Fig. 2).

### Morphological comparison between parental and offspring strains

**Figure 3:**
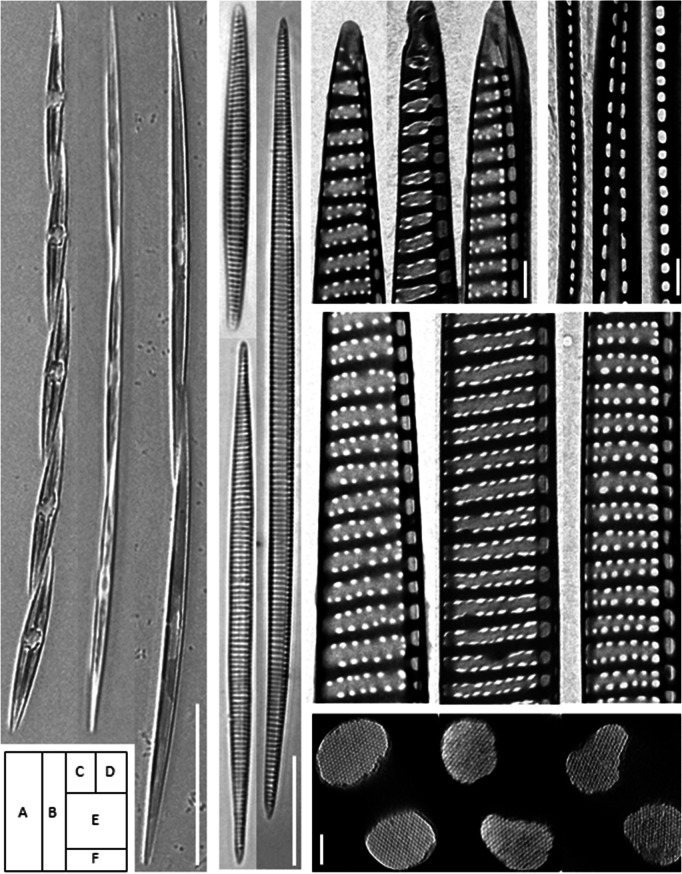
*Pseudo-nitzschia pungens* var. *pungens* valve shapes and frustules of parents and their offspring, light microscopy (**a**,**b**) and transmission electron microscopy (**c**–**e**). Chain formation of live cells (**a**), acid treated complete frustules (**b**), shape of cell tip (**c**), valvocopulae with one row of simple poroids of various sizes (**d**), internal valve view (**e**) and the mesh structure of each cell of poroid (**f**), in all, from the left, HY40E5 (clade I), Pnsb109 (clade III) and HYOFB6 (offspring). Scale bar = (**a**) 50 µm, (**b**) 20 µm, (**c**,**d**) 1 µm, (**e**) 5 µm, (**f**) 100 nm.

Except for the valve length, no distinct morphological features were observed between parent (HY38B2 or HY40E5) and offspring strains (HYOFA5, HYOFB6, HYOFB7 and HYOFE3). All *P. pungens* cells was spindle-like and linear in valve view (Fig. 3a,b), and the striae of all strains consisted of two rows of poroids (Fig. 3c,e). Both parent and offspring strains were identified as *P. pungens* var. *pungens* according to their valvocopulae which consisted of one row of circular or oval poroids (Fig. 3d). There was no significant difference (*p* = 0.05) in numbers and shapes of ultra-microstructures such as fibulae, striae, and poroids between parents and offspring strains (see the Supplementary Table S2). The mesh structures in poroids of all parent and offspring strains were microhexagon (Fig. 3f).

### Direct sequencing for the ITS2 of offspring strains

The ITS2 results from direct sequencing of the parent strains did not reveal any ambiguous peaks. The sequence of HY40E5 aligned 100% with all sequence from clade I^35,42^ strains available in NCBI. Similarly, the sequences of Pnsb109 were aligned 100% with clade III strains, from Korea (KJ146693, KJ146694, HYNG28C10 and HYGO29C11), Vietnam (DQ166533), Philippines (HYPP37F7) and Malaysia^43^. The sequences obtained from the offspring showed ambiguous peaks with nucleotides from both parents (clade I and III) at 12 positions (see the Fig. 5) of the ITS2 region.

### ITS2 secondary structure

**Figure 4:**
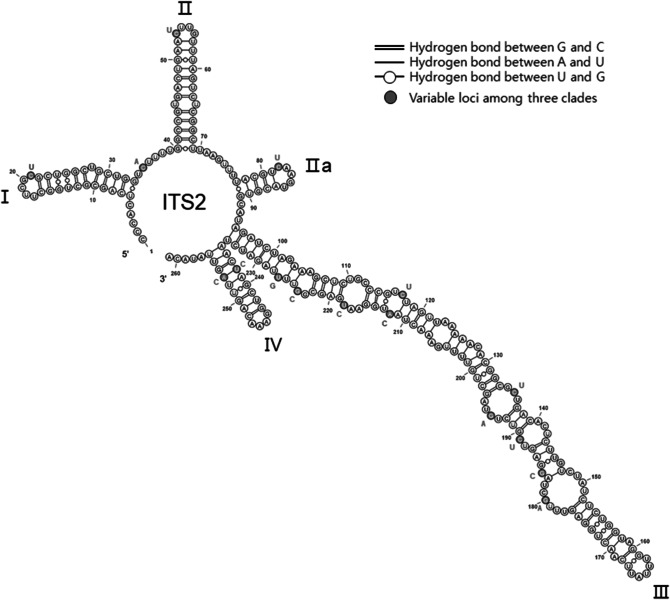
The proposed secondary structure of ITS2 transcripts of *Pseudo-nitzschia pungens* strains used in the mating experiments*.* Major helices are labelled from I to IV. The white circles are conserved loci for each clade and the gray circles indicate the variable loci. Colored letters indicate the substitutions (blue = clade II, red = clade III and purple = both clades I and III). There is no compensatory base pairs and hemi- compensatory base pairs among strains examined in this study.

The ITS2 secondary structure of *P. pungens* was predicted using direct sequencing results of strains of three clades. The structures were highly conserved, given four helices (I–IV) with a pseudo-helix, IIa (Fig. 4). Total 16 base changes were observed among three clades, but there compensatory base change (CBC) and hemi-compensatory base change (HCBC) between different clades.

### Genetic polymorphisms

**Figure 5:**
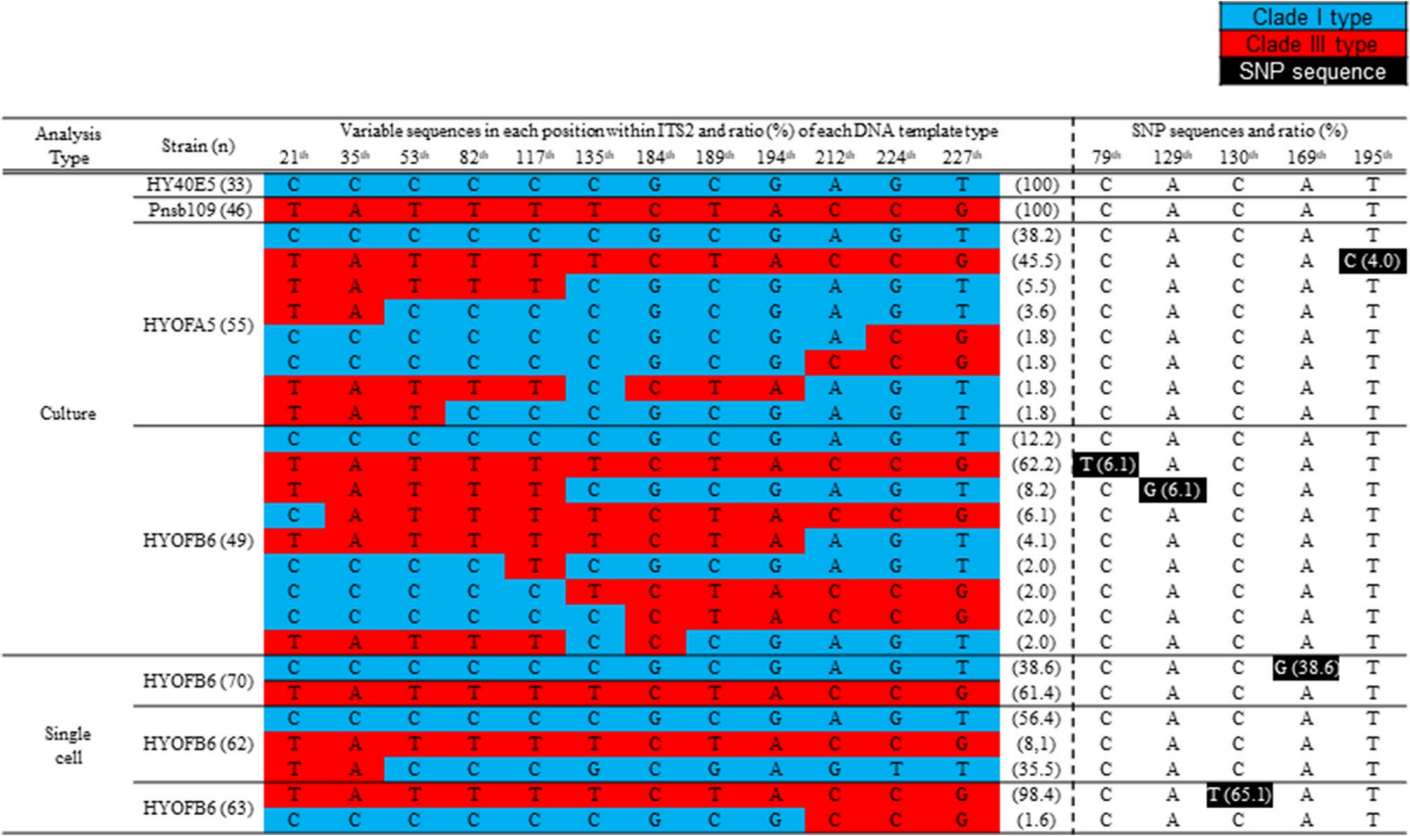
Summary of cloning results of parent and offspring cultures and single cells of offspring strain (HYOFB6). The variable sequences indicate the conserved sequences for each clade (blue; clade I and red; clade III). Single nucleotide polymorphism (SNP) sequences are indicated in black boxes. Rate of SNPs is written in parentheses next to each SNP sequence.

DNA templates of offspring sequenced after cloning confirmed that recombinant patterns were present in the ITS2 region (Fig. 5). The sequence variations and single nucleotide polymorphisms (SNPs) present in the ITS2 rDNA sequences are shown in Fig. 5. The sequences recovered from HYOFA5 were typical of clade I (38.2%), clade III (45.5%) and a hybrid type (16.3%). Sequences obtained from strain HYOFB6 were those of clade I (12.2%), clade III (62.2%) and a mixed type (25.6%) (Fig. 5). Results from strain HYOFB6 single cells revealed less diverse pattern, however recombinant patterns were also observed (Fig. 5). SNPs were detected at five positions in the ITS2 region of offspring strains. In cloning results of offspring mono-cultures, the HYOFA5 strain showed 4.0% SNPs at the 195th position, and HYOFB6 strain showed 6.1% of SNPs at the 79th and 129th positions in the ITS2 (Fig. 5). The two single cells showed 65.1% and 38.6% of SNPs at the 130th and 179th positions, respectively (Fig. 5).

## Discussion

The two classical mechanisms of reproductive isolation considered to lead to speciation have been designated as ‘prezygotic’ or ‘postzygotic’^49^. One of the main mechanisms of prezygotic isolation is “ecological isolation”, which includes a restricted distribution to different, or non-overlapping habitats, or ecological niches. Allopatric speciation is a representative concept for speciation^4^. In theory, the movement of marine plankton species is less restricted in the ocean because there are few geographical barriers in the ocean environment. From our results, sexual reproduction was possible among clade I populations, regardless of the geographical distance of their origin (Fig. 2). In between different clades, successful interbreeding was observed between clades I and II, and clades I and III, however it did not occur between clades II and III (Table 1). The three clades of *P. pungens* show quite distinct global distribution patterns^35^, however two expected hybrid zones (for clades I and II, and clades I and III) exist^40,48^. In previous work, however, hybrid zones for clade II and III were not found. Our results followed a ‘ring species’ concept^50,51^. Clade I, which are found worldwide, could hybridize with the other clades, but clade II and III, which are geographically isolated from one another, were unable to inter-breed. To date, there is no evidence that clade II and III can interbreed, but so far these clades cannot be distinguished by other species morphologically. Gene flow between them is still possible, as they both may interbreed with clade I. According to a previous study, clade II favours lower water temperature, whilst clade III favours higher water temperatures^42^. For hybrids to coexist in the Pacific-rim, clade II populations would have to cross the North Equatorial (warm) Current, or clade III would have to endure the Alaska and California (cold) currents. If prezygotic isolation occurs between clade II and III, their different ecological niches including distinct ecological habitats^35,43,48^ and eco-physiological traits^42^ might be the major cause. Successful migration and gene flow through sexual reproduction between populations is likely to prevent them from diverging too far and therefore becoming sexually incompatible. *P. pungens* was capable of sexual reproduction regardless of geographical distance, however a low probability or incompatibility of sexual reproduction between specific genetic populations may accelerate the species differentiation by continuous mutation, selection, and genetic drift.

Morphological differences is also considered as a main cause of prezygotic isolation^4^, however, it could not be considered in species of *Pseudo-nitzschia* because motile gametes are released and come into physical contact with the sessile gametes extracellularly. The morphological intraspecific diversity of *P. pungens* was also not related with the sexual incompatibility in our study. Successful interbreeding occurred between the different morphological varieties, *P. pungens* var. *pungens* (Pnpng1) and *P. pungens* var. *cingulata* (HY47B5), however it did not occur between Pnsb109 (*P. pungens* var. *pungens*) and HY47B5 (*P. pungens* var. *cingulata*) strains (Fig. 2; Table 1; Supplementary Fig. S1). Mating experiments using more morphological varieties isolated from diverse localities with diverse culturing conditions would provide a better understanding for the process of biological isolation and speciation in this species.

Previous studies have shown that clade III first diverged from clade I around 0.6 million years ago (Mya), and clades II and I differentiated between 0.2 and 0.8 Mya^36^. A greater genetic distance developed between clades II and III than between the other clades during this longer period of separation. The genetic distance between clades II and clade III was 3.1% (HY47B5–Pnsb109), I and II was 1.2% (Pnpng1–HY47B5), and I and III was 2.8% (HY40E5–Pnsb109 and HY38B2–Pnsb109) (Fig. 2; Supplementary Table S1). Among clade I strains, the genetic distances were less than 0.1%. The relationship between the genetic distance and intraspecific mating compatibility is not fully understood in diatoms, as successful interbreeding has been reported between strains with different genetic distances in diatom species. A genetic difference in the ITS rDNA of 0.6% (*P. multistriata*)^47^, 1.3% (*P. pungens*)^35^, 4.2% (*Eunotia bilunaris*)^52^ and 11.5–12.3% (*Eunotia bilunaris*)^53^ did not affect the ability of strains to interbreed. Indeed, hybrids can be obtained between different *Pseudo-nitzschia* species^54^. It has been argued that the secondary structure of the ITS2 provides important information in regards to sexual incompatibility^55^. The correlation between secondary structural features of the ITS2 and reproductive isolation has been demonstrated in many species^55,56^ in micro-algal species^57,58^ and ciliate species^59^, however, in our study, no correlation was observed. From our ITS2 secondary structure, compensatory base change (CBC) and hemi compensatory base change (HCBC) were not found between clades, and there were no large structural changes due to base changes (Fig. 4). Only clade II possessed an A → C and a U → C changes which induce a structural change in helix IV, but it also could not explain the sexual compatibilities between different clades. Additionally, the SNP positions of 79th, 129th and 130th ITS2 also did not produce any structural changes because they were hemi compensatory base changes, and the 169th and 195th SNP positions were located in loop region. Amato et al. have demonstrated that the ITS2 secondary structure indeed can be considered as a proxy for sexual compatibility, but it is hard to find a consistent correlation between mating compatibility and the ITS2 secondary structure in studies of diatoms. Because the reproductive isolation occurred if there were CBCs or HCBCs, but the reverse was not necessarily true^7^. These results indicate that there is no exact genetic distance threshold that corresponds to reproductive isolation in *Pseudo-nitzschia* species. However, only a very limited genetic area (only ITS2) has been taken into account, which requires further investigation for better understanding.

**Figure 6:**
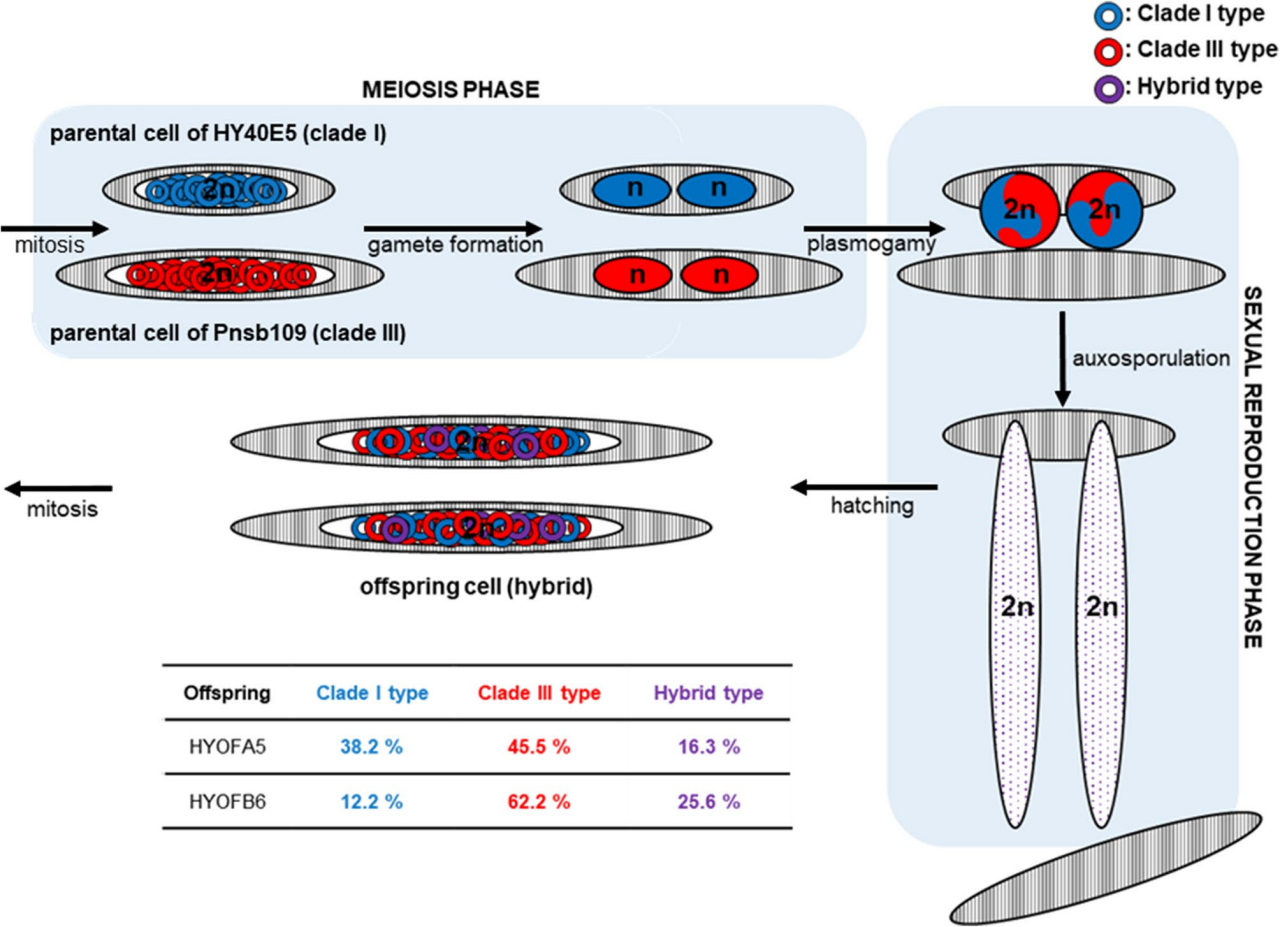
Schematic diagram of sexual reproduction in *Pseudo-nitzschia* and changes of the ITS2 gene copies after sexual reproduction. The ploidy levels (n or 2n) of cells of each phase were followed by diatom concept of Amato^60^, and approximate ITS2 gene copies of offspring strains are estimated from results of cloning PCR as shown in the accompanying Fig. 5.

No morphological intermediate was observed from our established offspring strains (Fig. 3; Supplementary Table S2), but genetic intermediates were confirmed. In regards to the causes of speciation in *Pseudo-nitzschia*, the hybrids may contribute to the adaptive variation in species survival^25,26^, and may even be the source of new recombinant species^27,28^. The rDNA encodes the ribosomal RNA that plays a major role in formation of ribosome and protein synthesis. The phenotypes and physiological characteristics are determined by distinct genes inherited from parents, and the morphological and/or physiological intermediates might be produced by genetic recombination in the F1 generation. *Pseudo-nitzschia* species inherit half (n) from each parents^60^. In our results, the offspring strains contained paternal and maternal rDNA copies of the ITS2, as well as gene copies of mixed type (Figs. 5, 6). The combinations of mixed type consisted of sequences of their parents only. Crossing-over is the exchange of chromosome segments between nonsister chromatids in meiosis. It creates new combinations of genes that are not found in either parent, contributing to genetic diversity^61^. The offspring having diverse gene types can have greater potential to adapt to various environments than their parents. In addition, single nucleotide polymorphisms (SNPs) were also found in several positions of the ITS2 (Fig. 5). In two single cells of offspring (HYOFB6), SNPs were found with high mutation ratios, at 38.6 and 65.1%, respectively (Fig. 5). In many species, SNPs occur every 200–500 base pairs, and are found in mostly noncoding regions^62,63^. SNPs are highly abundant but have slow mutation rates, and their generation via mutations may provide a significant pathway to generate novel genotypes with different haplotypes, thus retaining genetic diversity^64^. Interestingly, there is no distinct genetic variation within clade I and II groups, however clade III, which diverged from clade I, has more complex and diverse genetic variations within that group including genetically distinguished three sub-clades (IIIaa, IIIab and IIIb)^36,42,43^. Sexual reproductions that occurred between genetically different populations may indicate one mechanism for the formation of a unique genetic structure of this diatom species. Clade III is composed of three sub-clades^42^, and each sub-clade has the unique sequences similar to SNP in the ITS2 region (6th, 122th, and 211rd site) (Table S3). Moreover, sequence in the IIIab and IIIb sub-clades appeared the mixed pattern of IIIaa and clade I at two positions (184th and 189th site) (Table S3). Our findings suggest that sexual reproduction might lead to the SNP generation and gene recombination, resulting in production of novel sequence patterns.

Several morphological intermediate isolates of *P. pungens,* which seemed to be offspring generated between clades I and II, were found in the natural environment^40^, but expected hybrids of clades I and III have not been found. It is uncertain why these novel types of sequences have not been found in the field. This may be because this sexual phenomenon is very rare in nature, or it is only possible to generate hybrids under artificial laboratory conditions. Co-occurring blooms of clades I and III have been reported, however, hybrids were not observed at that time^48^. If the recombinants were not found despite frequent sexual phenomenon and co-occurrence in nature, ‘postzygotic isolation’ may also be occurring. Postzygotic isolation can happen due to several reasons; (1) hybrid unviability, and (2) hybrid sterility or breakdown^65^. Hybrid breakdown is more common in animal species. For example, horses and asses can easily be crossed and produce mules, however this hybrid is sterile. In comparison, our results of mating experiments showed that the offspring strains maintained reproductive ability, as they could successfully breed with the parent generation as well as with another offspring strains (Table 2). The viability of F1 generations to various environmental conditions has not yet been investigated. Fitness is one of the major criteria for interbreeding success^66^. Besides hybrid viability, interbreeding success is also regulated by various factors such as selective mating (gametic selection), frequency of bias reproduction, and different fertilities of parents^49^.

Sexual reproduction may allow for the maintenance of the genetic diversity of species via genetic recombination, and therefore the frequency of sexual reproduction may have a fundamental function in establishing the genetic structure of a population^23,67^. Diatom life history may follow three ‘cardinal points’ which are highly useful in systematics as well, especially in pennate diatoms^68^: (1) size of initial cells, (2) the upper limit of cell size for mating, (3) critical minimal cell size or in the case of species with a ‘closed life cycle’ the lower size limit cells can mate. According to other studies, the threshold of cell length of *P. pungens* was reported as 20–60% of the maximal cell (initial cell) length under laboratory conditions^22^, and > 62% in the field^31^. This may be an underestimation. From our results, the second cardinal point of *P. pungens* was at least > 66%, and offspring cells of *P. pungens* could mate after just a year (Fig. 1). According to field observations and growth models, sexual events of *Pseudo-nitzschia* species can occur every 2–3 years^24,69^, The cardinal points and decreasing rate of cell length are important factors which influence how frequently the hybrids may be potentially produced in natural systems.

We confirmed the successful interbreeding or lack of interbreeding between different clades of *P. pungens*, and established offspring strains of F1 generation from the mating experiments. Diverse novel ITS2 sequences of *P. pungens*, which were generated by genetic recombination and SNPs after sexual reproduction, were found. Sexual recombination along with mutations ultimately produces the high standing genetic variation observed in phytoplankton populations, in the form of nearly infinite numbers of genotypes within populations^70^. Such genetic polymorphisms also enhances the additive genetic variance in a population by bringing together fitness-increasing genetic variants^71^. Moreover, recombination after a longer phase of clonal propagation will release considerable hidden genetic variance within an asexually propagating population, producing novel phenotypes on which selection can act. Speciation by hybridization has been proposed in *Pseudo-nitzschia* genus^72^. Our study provided evidence of genetic polymorphisms due to sexual reproduction at the intra-specific level*.* In order to investigate the detailed population genetic structure in this species, more precise population analyses are needed using sensitive and diverse molecular markers such as microsatellites^73^. In order to better understand the possible role of hybrids in speciation and diversification in this genus, molecular techniques for the detection of hybrids in the field, the analysis of wider genetic region and subsequent rDNA monitoring will be helpful to resolve these questions.

## Methods

### Strain collection and culturing

*Pseudo-nitzschia pungens* strains belonging to the three recognized clades were either collected in the field or obtained from culture collections for sexual reproduction experiments (Supplementary Table S4). Five clade I strains were isolated from the Korean coasts (HY38B2 and HY40E5); Nanaimo island, Canada (HY40C3 and HY48D2); and from coast near the Cawthron Institute, New Zealand (Pnpng1). Three clade II strains (HY47B2, HY47B3 and HY47B5) were established from Puget Sound, Washington State of USA. One of three clade III strains were isolated from Santubong coast of Sarawak, Malaysia (Pnsb109) was obtained from a Bachok Marine Research Station, Malaysia; and the other two strains were isolated from Korean coasts (HY29B9 and HY48C9). Strains isolated from the field were established from single cells isolated from surface water samples by Pasteur pipette (Hilgenberg, Germany) under an inverted microscope (Model IX71, Olympus, Japan). Isolated cells were then washed with sterile seawater filtered GF/F filters (Whatman, USA), and transferred into individual 96-well plates filled with 200 μL of F/2 medium^74^. After 3–7 days, established monoclonal cells were transferred into a cell culture flask (SPL, Korea) containing 25 mL of sterile F/2 medium. All cultures were maintained at 20 °C with cool-white fluorescent lamp (12:12, light:dark photoperiod) and transferred every 20–30 days into 25 mL of new media before the experimental procedure began.

### Mating experiments and offspring strain establishment

Five milliliters of exponentially growing culture from each strain was inoculated into individual wells of 6-well plates and mixed with 5 mL of another *P. pungens* culture and incubated at 20 °C temperature. These ‘co-cultivation’ sets were grown in triplicate (Table 1). Additionally, several co-cultivation sets using randomly selected HY38B2, HY40E5, HY40C3, HY48D2, Pnpng1 and HY47B5 strains were performed in temperature conditions to investigate the effect of temperature on interbreeding between different clades – each triplicate set at 15, 20 and 25 °C. Plates were maintained under a 12:12 light:dark photoperiod with 80 μmol photons m^−2^ s^−1^ provided by cool-white fluorescent lamps for 30 days. When sexual reproduction was successful, that is, gamete and early-auxospore (zygote of diatom) were observed, the latter were isolated and transferred into new well plates for developing and hatching of auxospores. Once hatched, resultant offspring strains were maintained under the abovementioned conditions with F/2 medium. Intraclade offspring was not isolated.

### Sexual viability of offspring strains

To check the sexual viability of offspring strains, 5 mL of offspring cultures (HYOFA5, HYOFB6, HYOFB7 and HYOFE3) were immediately co-cultivated at a 1:1 ratio with a parental culture (either HY38B2 or HY40E5), and with another offspring culture and maintained using the above-mentioned conditions (at 20 °C only). Mating experiments were repeated 1 year later. This time delay ensured that all cultures reached their threshold size for sexual reproduction to occur^22,31^. All mating experiments were conducted in triplicate. In all mating experiments, the mating-type of each strain was determined by previous sexual reproduction experiment^22^. Throughout these experiments (from time of establishment through to the last mating experiment), the valve length of both parent and offspring strains were measured using and Eclipse Ci microscope (Nikon, Tokyo, Japan) at 1,000 × magnification. 15 cells for the offspring and 20 for parental strains were measured for morphometric analyses.

### Geographic and genetic distances

Geographic and genetic distances between parental *P. pungens* strains used in mating experiments under different temperatures were measured to evaluate potential reasons for reproductive isolation if sexual incompatibility was observed. Geographic distances were measured using the website Google Maps (https://map.google.com) and genetic distances were calculated in a Bioedit software (v. 7.1.3.0) using the ITS sequences. All ITS sequences were obtained from the PCR protocol described in below.

### Strain morphology

In order to examine and compare the fine scale structures of parents and offspring strains, 15 mL of each parent (HY40E5 and Pnsb109) and their offspring (HYOFB6) cultures were preserved with Lugol’s solution^74^ prior to light (LM) and transmission electron microscopy (TEM). The valve length and width of individual cells of each strain was measured using light microscopy (max. 1,000 × magnification) using an Eclipse Ci microscope equipped with a Lumenera Infinity 3 to 1 digital camera (Ottawa, Canada). Three milliliters of each acid treated^75^ sample were then placed on a formvar-coated copper grid and examined with an FEI Tecnai T20 transmission electron microscope (LaB6, Hillsboro, OR, USA). Images were taken using a 894 CCD camera (Gatan Inc., Pleasanton, CA, USA) and morphometric features analyzed using Image J software (v 1.44) (National Institutes of Health, USA)^76^. The ultra-microstructures (number of fibulae, striae and poroids) were counted as per the standard published methods^77,78^. The cingular band structures of parent and offspring strains were recorded as well to determine the varieties of *P. pungens*. Statistical analyses on all morphometric data were carried out using SPSS software (v. 8.0). One-way analyses of variance (ANOVA) were carried out to compare morphometric features between the parent and offspring strains. Tukey and Duncan’s tests were performed for significance verification (*p* = 0.05) with 1,000 replicates. For all morphological comparisons, twenty (for parent strains) and fifteen individual cells were analyzed.

### PCR and direct sequencing

Fifteen milliliters of each parent (HY40E5 and Pnsb109) and randomly selected two offspring (HYOFA5 and HYOFB6) cultures were harvested in the stationary growth phase and pelleted by centrifugation at 2,000*g* (4 °C, 15 min). The genomic DNA of each sample was extracted using a DNeasy Plant Mini Kit (Qiagen, Valencia, CA, USA) using the manufacturer’s protocols. The ITS2 rDNA region was amplified using the forward primer; PnITSF (ACT TTC AGC GGT GGA TGT CTA) and the reverse primer; PnITSR (CTT GAT CTG AGA TCC GGA ATT) following the PCR protocols from a previous study^42^. Amplicons were separated on 1.3% w/v agarose gel and purified using the Qiaquick PCR purification kit (Qiagen, Hilden, Germany). The PCR products were then directly sequenced with a Big DyeTM Terminator Cycle Sequencing Ready Reaction Kit (Applied Biosystems, Foster City, CA, USA). Sequences of parents and offspring strains were aligned and compared with each other using Bioedit software (v 7.1.3.0).

### rRNA secondary structure

The ITS2 secondary structure of parent strains of *P. pungens* used for mating experiment was predicted by homology modeling^79^, and the structure was visualized using VARNA^80^. The ITS2 region was identified using hidden Markov models^81^. The compensatory base changes were determined with 4SALE software^82^.

### Cloning PCR

PCR products were cloned and sequenced in order to confirm the genetic recombination in the ITS2 rDNA regions of the offspring. To construct the clone libraries, we used DNA fragments of the ITS2 obtained from the PCR products. Other DNA fragments of the ITS2 rDNA were also prepared from a nested single cell PCR using the offspring culture (HYOFB6) to confirm the genetic recombination in individual cells. Five individual cells of HYOFB6 were randomly isolated and the genomic DNA of each cell was extracted using the direct extraction method^83^. Nested single cell PCR was conducted with the protocols from a previous study^42^. Unamplified PCR products were sorted using agarose gel electrophoresis. Finally, a total of seven (two for each parent cultures, two for offspring cultures and three for offspring single cells) samples of DNA fragments were prepared and cloned into the plasmid vector pCR2.1 using the TOPO TA cloning kit (Invitrogen, CA, USA). The plasmid vectors were transformed into One Shot1TOP10 Chemically Competent *E. coli* (Invitrogen, CA, USA) following the manufacturer’s protocol. Recombinants were cultured in L1 medium and incubated at 37 °C for 1 h, then spread onto selective L1 agar (containing 50 μg mL^−1^ of ampicillin). After 24 h, between 33 to 70 recombinant colonies of each sample were picked and transferred into 1.5 mL sterile tubes (Axygen Sciences, CA, USA) containing 50 μL MilliQ water. PCR was performed to analyze the inserted DNA fragments. The PCR contained PCR buffer, 0.3 μM of universal primers (M13F and M13R), a dNTP mixture (0.25 μM of each dNTP), 0.2 units Takara Ex Taq polymerase (TaKaRa, Osaka, Japan), and PCR-grade water (Sigma-Aldrich, MO, USA). The PCR cycle was 95 °C for 10 min, then 39 cycles at 95 °C for 20 s, 55 °C for 30 s, 72 °C for 1 min, and a final extension at 72 °C for 10 min. The PCR products were then directly sequenced using the method described earlier, and the obtained sequences aligned and edited using BioEdit software version 7.1.3.0. From the cloned sequences, the positions of SNPs were determined, in cases in which the ratio of mutation was over one percent at each position of the cloned DNA template^63^.

## Supplementary information

Supplementary information
