# Supplementary material for: Sexual reproduction and genetic polymorphism within the cosmopolitan marine diatom Pseudo-nitzschia pungens
Source: Sci Rep. 2020 Jun 30;10:10653. doi: 10.1038/s41598-020-67547-9 (PMC7326933; doi:10.1038/s41598-020-67547-9)
Supplement: Supplementary file 1 — Supplementary information [file 41598_2020_67547_MOESM1_ESM.pdf]

Sexual Reproduction and Genetic Polymorphism within the Cosmopolitan Marine Diatom *Pseudo-nitzschia pungens*

Jin Ho Kim[1, 2, 3], Penelope Ajani[4], Shauna A. Murray[4], Joo-Hwan Kim[1], Hong Chang Lim[5], Sing Tung Teng[6], Po Teen Lim[7], Myung-Soo Han[1,]* and Bum Soo Park[1,8,]*

[1]Department of Life Science, College of Natural Sciences, Hanyang University, Seoul 04763, Republic of Korea, [2]Risk Assessment Research Center, Korea Institute of Ocean Science and Technology, Geoje 53201, Republic of Korea, [3]DNA Analysis Division, National Forensic Service, Seoul 158-707, Republic of Korea, [4]Climate Change Cluster, University of Technology Sydney 2007, Australia, [5]Regal City College, 93150 Kuching, Sarawak, Malaysia, [6]Faculty of Research Science and Technology, University Malaysia Sarawak, Kota Samarahan 94300, Malaysia, [7]Bachok Marine Research Station, Institute of Ocean and Earth Sciences, University of Malaya, Bachok, Kelantan 16020, Malaysia, [8]Marine Ecosystem Research Center, Korea Institute of Ocean Science and Technology, Busan 49111, Republic of Korea

* Co-Corresponding authors;

Myung-Soo Han: hanms@hanyang.ac.kr, Tel: +82-2-2220-0956; Fax: +82-2-2220-1171 and

Bum Soo Park: parkbs@kiost.ac.kr, Tel: +82-51-664-3336

Table S1. The genetic distances calculated with ITS sequences among three clades of *Pseudo-nitzschia pungens*. The dark grey area indicates clade I, light grey clade II, and the clear area indicates clade III strains, respectively. The strains used for mating experiments in this study are shown with solid boxes, and the genetic distances between these strains are shown in the dotted boxes. The genetic information of *P. pungens* strains used in this table is obtained from the previous study[38].

		Pnpng1	HY40E5	HY40C3	HY48D2	HY38B2	EC110836B6	AM778785	AM778777	AM778803	AM778815	AM778817	AM778787	AM778786	AM778788	AM778769	AM778775	AM778812	FN823051	HY48B5	NWFCS356	AM778795	AM778796	AM778804	AM778811	PnSB109	NG101029F5	EC110836C7	DQ166533	DQ062665	EC110836E10	PP111037F8	PP111037F9
Clade I	Pnpng1																																
	HY40E5	0.000																															
	HY40C3	0.000	0.000																														
	HY48D2	0.000	0.001	0.001																													
	HY38B2	0.000	0.000	0.000	0.001																												
	EC110836B6	0.000	0.000	0.000	0.001	0.000																											
	AM778785	0.000	0.000	0.000	0.001	0.000	0.000																										
	AM778777	0.000	0.000	0.000	0.001	0.000	0.000	0.000																									
	AM778803	0.000	0.000	0.000	0.001	0.000	0.000	0.000	0.000																								
	AM778815	0.000	0.000	0.000	0.001	0.000	0.000	0.000	0.000	0.000																							
	AM778817	0.000	0.000	0.000	0.001	0.000	0.000	0.000	0.000	0.000	0.000																						
	AM778787	0.000	0.000	0.000	0.001	0.000	0.000	0.000	0.000	0.000	0.000	0.000																					
	AM778786	0.000	0.000	0.000	0.001	0.000	0.000	0.000	0.000	0.000	0.000	0.000	0.000																				
	AM778788	0.000	0.000	0.000	0.001	0.000	0.000	0.000	0.000	0.000	0.000	0.000	0.000	0.000																			
	AM778769	0.000	0.000	0.000	0.001	0.000	0.000	0.000	0.000	0.000	0.000	0.000	0.000	0.000	0.000																		
	AM778775	0.000	0.000	0.000	0.001	0.000	0.000	0.000	0.000	0.000	0.000	0.000	0.000	0.000	0.000	0.000																	
	AM778812	0.000	0.000	0.000	0.001	0.000	0.000	0.000	0.000	0.000	0.000	0.000	0.000	0.000	0.000	0.000	0.000																
	FN823051	0.000	0.000	0.000	0.001	0.000	0.000	0.000	0.000	0.000	0.000	0.000	0.000	0.000	0.000	0.000	0.000	0.000															
clade II	HY47B5	0.012	0.012	0.012	0.013	0.012	0.012	0.012	0.012	0.012	0.012	0.012	0.012	0.012	0.012	0.012	0.012	0.012	0.012														
	NWFCS356	0.012	0.012	0.012	0.013	0.012	0.012	0.012	0.012	0.012	0.012	0.012	0.012	0.012	0.012	0.012	0.012	0.012	0.012	0.000													
	AM778795	0.012	0.012	0.012	0.013	0.012	0.012	0.012	0.012	0.012	0.012	0.012	0.012	0.012	0.012	0.012	0.012	0.012	0.012	0.000	0.000												
	AM778796	0.012	0.012	0.012	0.013	0.012	0.012	0.012	0.012	0.012	0.012	0.012	0.012	0.012	0.012	0.012	0.012	0.012	0.012	0.000	0.000	0.000											
	AM778804	0.012	0.012	0.012	0.013	0.012	0.012	0.012	0.012	0.012	0.012	0.012	0.012	0.012	0.012	0.012	0.012	0.012	0.012	0.000	0.000	0.000	0.000										
	AM778811	0.012	0.012	0.012	0.013	0.012	0.012	0.012	0.012	0.012	0.012	0.012	0.012	0.012	0.012	0.012	0.012	0.012	0.012	0.000	0.000	0.000	0.000	0.000									
clade III	PnSB109	0.028	0.028	0.028	0.030	0.028	0.028	0.028	0.028	0.028	0.028	0.028	0.028	0.028	0.028	0.028	0.028	0.028	0.028	0.031	0.031	0.031	0.031	0.031	0.031								
	NG101029F5	0.028	0.028	0.028	0.030	0.028	0.028	0.028	0.028	0.028	0.028	0.028	0.028	0.028	0.028	0.028	0.028	0.028	0.028	0.031	0.031	0.031	0.031	0.031	0.031	0.000							
	EC110836C7	0.028	0.028	0.028	0.030	0.028	0.028	0.028	0.028	0.028	0.028	0.028	0.028	0.028	0.028	0.028	0.028	0.028	0.028	0.031	0.031	0.031	0.031	0.031	0.031	0.000	0.000						
	DQ166533	0.030	0.030	0.030	0.031	0.030	0.030	0.030	0.030	0.030	0.030	0.030	0.030	0.030	0.030	0.030	0.030	0.030	0.030	0.033	0.033	0.033	0.033	0.033	0.033	0.001	0.001	0.001					
	DQ062665	0.031	0.031	0.031	0.033	0.031	0.031	0.031	0.031	0.031	0.031	0.031	0.031	0.031	0.031	0.031	0.031	0.031	0.031	0.034	0.034	0.034	0.034	0.034	0.034	0.009	0.009	0.009	0.010				
	EC110836E10	0.028	0.028	0.028	0.030	0.028	0.028	0.028	0.028	0.028	0.028	0.028	0.028	0.028	0.028	0.028	0.028	0.028	0.028	0.031	0.031	0.031	0.031	0.031	0.031	0.000	0.000	0.000	0.001	0.009			
	PP111037F8	0.031	0.031	0.031	0.033	0.031	0.031	0.031	0.031	0.031	0.031	0.031	0.031	0.031	0.031	0.031	0.031	0.031	0.031	0.034	0.034	0.034	0.034	0.034	0.034	0.009	0.009	0.009	0.010	0.003	0.009		
	PP111037F9	0.030	0.030	0.030	0.031	0.030	0.030	0.030	0.030	0.030	0.030	0.030	0.030	0.030	0.030	0.030	0.030	0.030	0.030	0.033	0.033	0.033	0.033	0.033	0.033	0.010	0.010	0.010	0.012	0.004	0.010	0.004	

Table S2. Morphometric data (average ± SD) of each parent and offspring strain of *Pseudo-nitzschia pungens*

Strain (n)	Genotype	Generation	Fibulae (in 10μm)	Striae (in 10μm)	Poroids (in 1μm)	Remark
HY40E5 (20)	Clade I	parent	12.63 ± 1.00	11.92 ± 0.73	3.03 ± 0.17	*P. pungens* var. *pungens*
Pnsb109 (20)	Clade III	parent	13.80 ± 0.93	13.36 ± 0.64	3.38 ± 0.24	
HYOFB6 (15)	n. d	offspring	12.96 ± 1.23	12.36 ± 1.35	2.84 ± 0.22	

n; measured individual cell number,

n.d; genotypes were not determined due to mixed sequences.

There is no significant morphological difference among three strains in ANOVA test ($p = 0.05$)

Table S3. Variable sequences of each sub-clade of clade III in ITS2 region.

Clade	Sub-clade	Variable sequences at each locus of ITS2														
		6th	21rd	35th	53th	82th	117th	122th	135th	184th	189st	194th	211rd	212th	224th	227th
III	IIIaa	C	T	A	T	T	T	G	T	C	T	A	G	C	C	G
	IIIab	C	T	A	T	T	T	G	T	C	C	A	A	C	C	G
	IIIb	T	T	A	T	T	T	A	T	G	C	A	G	C	C	G
I	n	C	C	C	C	C	C	G	C	G	C	G	G	A	G	T

Table is organized on the data of the previous study[38].

The ITS2 sequences varied in shading locus within clade III.

n; clade I has no sub-clade.

Table S4. *Pseudo-nitzschia* clones used in this study including species, strain, clade, origin, data of isolation and Genbank accession number.

Species	Strain	Clade	Origin	Isolated date
P. pungens var. *pungens*	HY38B2	I	Korea, Jeju Island, Port Jeju	2012 / 10
var. *pungens*	HY40E5	I	Korea, Geojae Island, Jangmok	2014 / 10
var. *pungens*	HY40C3	I	Canada, Nanaimo Island, Inner Harbour	2013 / 10
var. *pungens*	HY48D2	I	Canada, Nanaimo island, Inner Harbor	2013 / 10
var. *pungens*	Pnpng1	I	New Zealand, Nelson, near Cawthron Institute	2014 / 09
var. *cingulata*	HY47B2	II	USA, Washington State, Puget Sound	2014 / 06
var. *cingulata*	HY47B3	II	USA, Washington State, Puget Sound	2014 / 06
var. *cingulata*	HY47B5	II	USA, Washington State, Puget Sound	2014 / 06
var. *pungens*	Pnsb109	III	Malaysia, Kuching, Santubong	2014 / 02
var. *pungens*	HY29B9	III	Korea, Namhae Island, Namhae	2013 / 08
var. *pungens*	HY48C9	III	Korea, Masan Bay	2014 / 08
var. *pungens*	HYOFA5	n. d	Offspring of HY40E5 and Pnsb109	2015 / 06
var. *pungens*	HYOFB6	n. d	Offspring of HY40E5 and Pnsb109	2015 / 06
var. *pungens*	HYOFB7	n. d	Offspring of HY40E5 and Pnsb109	2015 / 06
var. *pungens*	HYOFE3	n. d	Offspring of HY40E5 and Pnsb109	2015 / 06

n.d; genotypes were not determined due to mixed sequences.

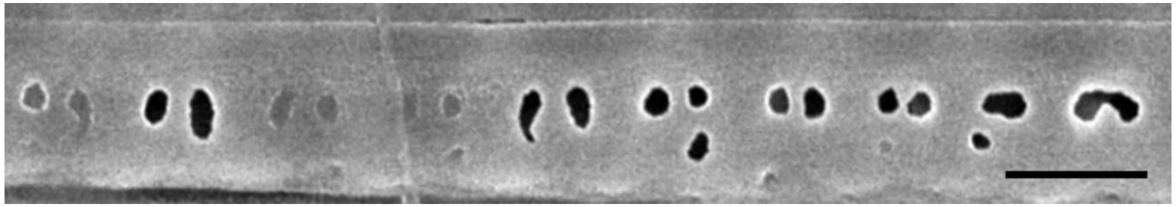

Fig. S1. Transmission electron microscope image of the valvocopulae of *Pseudo-nitzschia pungens* var. *cingulata* (HY47B5 strain) showing square structures consisting of 2 - 4 poroids. Scale bar: 1μm.
